# Competition on robust deep learning

**DOI:** 10.1093/nsr/nwad087

**Published:** 2023-04-07

**Authors:** Yinpeng Dong, Chang Liu, Wenzhao Xiang, Hang Su, Jun Zhu

**Affiliations:** Department of Computer Science and Technology, Institute for AI, Tsinghua-Bosch Joint ML Center, THBI Lab, BNRist Center, Tsinghua University, China; Institute of Image Communication and Networks Engineering in the Department of Electronic Engineering (EE), Shanghai Jiao Tong University, China; Key Laboratory of Intelligent Information Processing of Chinese Academy of Sciences (CAS), Institute of Computing Technology, CAS, China; Peng Cheng Laboratory, China; Department of Computer Science and Technology, Institute for AI, Tsinghua-Bosch Joint ML Center, THBI Lab, BNRist Center, Tsinghua University, China; Peng Cheng Laboratory, China; Pazhou Laboratory (Huangpu), China; Department of Computer Science and Technology, Institute for AI, Tsinghua-Bosch Joint ML Center, THBI Lab, BNRist Center, Tsinghua University, China; Peng Cheng Laboratory, China; Pazhou Laboratory (Huangpu), China

**Keywords:** robustness, adversarial example, adversarial training, deep learning

## Abstract

This perspective paper proposes a new adversarial training method based on large-scale pre-trained models to achieve state-of-the-art adversarial robustness on ImageNet.

## PROBLEM

In recent years, the rapid development of artificial intelligence (AI) technology, especially machine learning and deep learning, is profoundly changing human production and lifestyle. In various fields, such as robotics, face recognition, autonomous driving and healthcare, AI is playing an important role. However, although AI is promoting the technological revolution and industrial progress, its security risks are often overlooked. Previous studies have found that the well-performing deep learning models are extremely vulnerable to *adversarial examples* [1–3]. The adversarial examples are crafted by applying small, human-imperceptible perturbations to natural examples, but can mislead deep learning models to make wrong predictions. The vulnerability of deep learning models to adversarial examples can raise security and safety threats to various real-world applications.

In order to improve the robustness of deep learning models towards safe and reliable artificial intelligence, researchers have proposed various defense methods in recent years. However, the performance of the current defenses is still far from satisfactory. Adversarial robustness is often achieved at the cost of worse model accuracy on natural examples. Furthermore, there is a significant robust generalization gap. More importantly, most works focus on smaller datasets (e.g. CIFAR) to build robust models, while few works consider adversarial robustness on more practical large-scale datasets, such as ImageNet. Therefore, it is hard to assess the progress of adversarial robustness in real-world applications that often involve more realistic datasets.

To bridge this gap, we organized the *Competition on Robust Deep Learning*. This competition focuses on adversarial robustness on the ImageNet dataset [4], aiming to facilitate the development of more effective adversarial defense techniques at scale. In addition, with the development of different vision model architectures and pre-training techniques, the robustness of the model has also been improved. This competition also aims to explore efficient network architectures and training techniques for model robustness. Specifically, this competition adopts the ImageNet dataset that is widely used in academia, and uses typical adversarial attack algorithms to calculate the accuracy of the model under attack for evaluation. Below we introduce the detailed competition rules.

### Competition rules

#### Dataset

The competition adopts the ImageNet dataset, which contains ∼13 M images of 1000 classes. The competitors can train their robust models on additional datasets, such as ImageNet-21K. We evaluate the performance of submitted models on the ImageNet validation set in the first round as the public leaderboard. We have a private dataset compatible with ImageNet to provide the evaluation of each team at the end of the first round. In the final round, each team can submit a dataset for evaluation and the final evaluation will be conducted on a sampled dataset from all teams.

#### Evaluation metric

Given a test set of *N* images *X* = {*x*_1_, *x*_2_, …, *x_N_*}, we compute the robust accuracy under each perturbation budget ε as


(1) \begin{eqnarray*} \mathrm{Score}(\epsilon )&=&\frac{1}{N}\sum _{i=1}^{N}\min _{a\in A}\\ &&\times \, \mathbf {1}[f(a(x_i))=y_i],\\ \end{eqnarray*}

where *f*( · ) is the classification model submitted by a team, *A* is a set of different attack algorithms, *a* is a specific attack, *y_i_* is the corresponding ground-truth label of *x_i_* and **1**( · ) is the indicator function. After we have a score under ε, we compute the overall score of the submission as


(2) \begin{eqnarray*} \mathrm{Score}=\frac{1}{4}\sum _{\epsilon \in [0,2,4,8]}\mathrm{Score}(\epsilon ). \end{eqnarray*}

The final score is averaged over four different ε, including the clean accuracy as ε = 0. The evaluation metric is designed to reflect the trade-off between clean and robust accuracies.

#### Platform

We encourage the competitors to use the adversarial robustness platform ARES [5] to build and evaluate their models. However, they are not limited to this platform.

#### Additional restrictions

First, the model size should be smaller than 350 M. Second, the model should not have gradient obfuscation modules. This is because we mainly adopt gradient-based attacks to evaluate the robustness of each model. Gradient obfuscation can prevent gradient-based attacks to generate adversarial examples, but the model can be deceived by adaptive attacks. As we cannot automatically design an adaptive attack for each defense, we prohibit this type of defense.

## ALGORITHM

The proposed method is an efficient adversarial training algorithm based on pre-trained models. Adversarial training is one of the most effective defense methods to adversarial examples. It augments training data $\boldsymbol {x}$ with adversarial examples $\hat{\boldsymbol {x}}$, which can be formulated as a min-max optimization problem:


(3) \begin{eqnarray*} \min _{\boldsymbol {\theta }} \mathbb {E}_{(\boldsymbol {x},y) \sim \mathcal {D}} \max _{\hat{\boldsymbol {x}}:\Vert \hat{\boldsymbol {x}}-\boldsymbol {x}\Vert _p\le \epsilon } \mathcal {L}(\hat{\boldsymbol {x}},y;\boldsymbol {\theta }). \end{eqnarray*}

Here ε denotes the perturbation budget, ‖ · ‖_*p*_ is the *L_p_*-norm operation, $\mathcal {L}$ denotes the loss function and $\boldsymbol {\theta }$ denotes the weights of a classifier. The inner maximization can be solved by projected gradient descent (PGD) [6].

**Figure 1.**
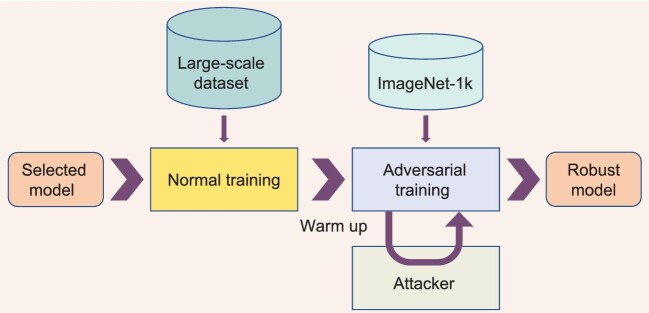
The training framework of the proposed method.

However, the training process of adversarial training requires high computational cost, which limits its application to large vision models. Motivated by the recent tendency to utilize large pre-trained models for downstream

applications in specific domains, it is proposed to fine-tune the models pre-trained on the ImageNet-21K dataset for adversarial robustness. The intuition behind fine-tuning the large pre-trained models is that parts of the feature extractors in pre-trained models are reusable during the fine-tuning process, which greatly accelerates the fine-tuning process. In spite of the efficiency of this method, the fine-tuning process experiences catastrophic forgetting on an upstream task, i.e. performance drop on clean samples. To mitigate this phenomenon, a warm-up process is brought in front of the adversarial training process. To be specific, the perturbation budget of the adversarial examples is linearly increased until epoch 5, and the final perturbation budget is set as 4/255. The training framework is shown in Fig. 1.

As a benefit of the efficiency of this method, it becomes possible to conduct comprehensive analysis on adversarial robustness from different aspects, such as model architectures and training strategies. For architectures, mainstream models (including ResNet, ConvNet in CNNs and ViT, DeiT, Swin in Transformers) as well as different model sizes are analyzed. For training strategies, data augmentations, regularization, weight averaging, PGD attacker and pre-training are analyzed.

The best robust model is trained based on Swin Transformer Large with input size 384 (the input images will be resized to 384). To prevent overfitting to the training data, data augmentations (including RandAugment and Mixup) and regularization (including Label Smoothing) are applied to the training process. To make the local optimal flatter, the exponential moving average (EMA) is applied to the training process. For better balance between different perturbation budgets, the PGD attacker with epsilon 4/255, step 3 and step size 8/3/255 are applied to generate adversarial examples. To accelerate the training process, the pre-trained Swin Large model in the official implementation is applied as the initialization of the training process.

## EVALUATION

The best model is obtained with a large amount of experiments from two aspects: model architectures and training strategies.

### Model architectures

First, different models (including ResNet, ConvNet in CNNs and ViT, DeiT, Swin in Transformers) are adversarially trained with the PGD attacker, which adopts the setting with epsilon 4/255, step 3 and step size 8/3/255. The training strategies are mostly aligned except for ResNets, because ResNets can quickly converge to the local optimal. The training processes of some models, such as ConvNet Small (ConvNetS) and DeiT Base (DeiTB), are stopped early for time limit, which are compared with Swin Transformer Small (SwinS) with the same training epochs. We find that Swin Transformers are more robust than other models. Detailed results are shown in the online supplementary material.

Second, the effect of different model sizes (including Small, Base, Large and Large384) is analyzed based on SwinS, which performs best among different architectures. Swin Transformer Large (SwinL) has 187M parameters, which satisfies the competition rules.

### Training strategies

The effect of different training strategies is analyzed based on SwinS. First, adversarial training also suffers from overfitting, which can be mitigated by data augmentations and regularization methods. Besides, a flatter optimal point has better robustness, which can be obtained by a weight averaging method, such as EMA. Because of the time limit, a detailed analysis of each training strategy is not provided. The baseline training setting contains no augmentations, regularization and weight averaging, while the strengthened one contains RandAugment, Mixup, Label Smoothing and EMA. We find that adversarial robustness is significantly improved with a combination of all these training strategies.

Second, there exists a trade-off between clean accuracy and adversarial robustness. To obtain a better score based on the competition rules, two settings for the PGD attacker are analyzed, with the former epsilon as 4/255 and the latter one as 8/255. We find that adversarial training with an 8/255 perturbation budget has better performance on eps8 evaluation, but that its performance on clean examples and eps2 adversarial examples drops more rapidly. Therefore, the final setting of epsilon for the PGD attacker is 4/255.

Third, adversarial training requires much computational cost, which can be improved by utilizing pre-trained models as the initialization for adversarial training. Among different pre-training methods, large-scale dataset pre-training (e.g. 21K pre-training) best matches this task, because the loss functions are consistent. The SwinS model trained from scratch and that trained from a 21K pre-trained initialization are compared. We find that fine-tuning the 21K pre-trained model has competitive robustness compared with training from scratch, and that the convergence time of the former is much faster than the latter.

## FUTURE RESEARCH

First, there is still a trade-off between model accuracy and robustness. Although we have witnessed a significant performance improvement in model robustness with advanced model architectures and training techniques, the clean accuracy of the model still drops. We found that using a large model size can improve model accuracy and robustness at the same time. Therefore, it requires future investigation to determine how to alleviate the trade-off.

Second, the training speed is still low. As adversarial training requires gradient backpropagation to generate adversarial examples, it is about 3–5 times slower than normal training. With the increased sizes of the model and dataset, performing adversarial training is more computationally expensive. Therefore, it also requires acceleration techniques for adversarial training.

Third, although this competition mainly focuses on adversarial robustness of deep learning models, these models are also vulnerable to natural distribution shifts, such as common corruptions [7], viewpoint changes [8], etc. Adversarially trained models may not generalize well to these distribution shifts, as shown in ref. [8]. There still lacks a deeper understanding of the relationship between different aspects of model robustness, which requires further exploration.

## Supplementary Material

nwad087_Supplemental_File
