# Supplementary material for: Competition on robust deep learning
Source: Natl Sci Rev. 2023 Apr 7;10(6):nwad087. doi: 10.1093/nsr/nwad087 (PMC10257479; doi:10.1093/nsr/nwad087)
Supplement: nwad087_Supplemental_File [file nwad087_supplemental_file.pdf]

Appendix

Table 1. Adversarial robustness on different models

Model	clean	eps2[a]	eps4[a]	eps8[a]
ResNet50[b]	65.9	52.2	37.8	15.7
ResNet101[b]	69.2	58.5	45.0	20.8
ConvNextS[c]	72.8	64.1	53.7	29.2
ViTS	69.6	59.0	46.4	19.4
ViTB	73.2	63.6	50.9	25.1
DeiTB[c]	72.7	63.3	51.2	26.6
SwinS[c]	73.6	65.6	55.2	31.4
SwinS	75.7	67.4	57.2	33.4
SwinB	**76.2**	**68.7**	**58.7**	**35.3**

[a]The notation epsn means epsilon n/255.
[b]Different training setting for ResNet.
[c]Training is stopped in epoch 200 because of time limit.

Table 2. Adversarial robustness on different model sizes

Model	clean	eps2	eps4	eps8
SwinS	75.7	67.4	57.2	33.4
SwinB	76.2	68.7	58.7	35.3
SwinL	78.9	70.9	61.4	36.7
SwinL384[a]	**80.2**	**73.4**	**62.9**	**37.7**

[a]The size of the input images is 384.

Table 3. Adversarial robustness on different training strategies

Model	clean	eps2	eps4	eps8
Baseline	72.4	61.8	48.7	23.0
Strengthened	**75.7**	**67.4**	**57.2**	**33.4**

Detailed results

Model Architectures. We train models with different architectures, including ResNet, ConvNext in CNNs and ViT, DeiT, Swin in Transformers. The adversarially-trained models are testes with PGD100, and the detailed results are shown in Tab. 1. It is shown that Swin Transformers are more robust than the others. The most robust model is SwinB, obtaining classification accuracy of 76.2% on clean samples, 68.7% on adversarial examples with epsilon 2/255, 58.7% on adversarial examples with epsilon 4/255, and 35.3% on adversarial examples with epsilon 8/255. That is because Swin Transformer has better model design, including patchified input images, enlarged kernel size, and reduced activation and normalization layers.

Model Size. We also conduct experiments by gradually increasing model size. The detailed results are shown in Tab. 2. It is shown that models with larger size have better performance on adversarial robustness. The model SwinL with 384 input resolution obtains the best performance as 80.2% on clean samples, 73.4% on on adversarial examples with epsilon 2/255, 62.9% on adversarial examples with epsilon 4/255, and 37.7% on adversarial examples with epsilon 8/255. It reveals that large model size means large model capacity to fit the distribution of adversarial examples.

Training Strategies. The effect of training strategies is analyzed based on SwinS. The baseline training setting contains no augmentations, regularization and weight averaging, while the strengthened one contains RandAugment, Mixup, Label Smoothing and EMA. Training strategies. Detailed results are shown in Tab. 3. It is shown that the strengthened model obtains the best results as 75.7% on clean samples, 67.4% on on adversarial examples with epsilon 2/255, 57.2% on adversarial examples with epsilon 4/255, and 33.4% on adversarial examples with epsilon 8/255. It means that adversarial training also suffers from overfitting, which requires much more training strategies to improve the final performance.

Epsilon Setting. To obtain a better score based on the competition rules, two settings for PGD attacker are analyzed, with the former epsilon as 4/255 and the latter one as 8/255. Detailed results are shown in Tab. 4. It is shown that the model trained with epsilon 4/255 has better performance on clean samples, and adversarial examples with epsilon 2/255 and 4/255.

Pre-training. Finally, we analyze the effects of model pre-training, and detailed results are shown in Tab. 5. To exhibit the efficiency of this method, the loss and accuracy in the training pro-

Table 4. Adversarial robustness on different perturbation budgets

Perturbation	clean	eps2	eps4	eps8
eps4	**73.6**	**65.6**	**55.2**	31.4
eps8	67.0	60.9	54.6	**38.1**

Table 5. Adversarial robustness on different model initialization

Method	clean	eps2	eps4	eps8
From Scratch	76.2	68.6	**58.6**	**35.2**
From 21K	**77.7**	**69.0**	57.7	32.0

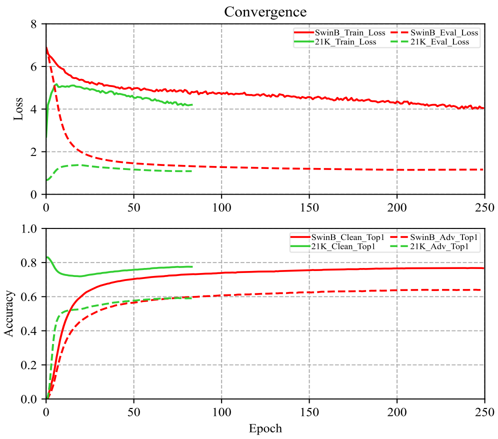

Figure 2. Training curves of vanilla AT and 21K-pre-training.

cess are visualized in Fig. 2. It is shown that the model trained from 21K pre-trained initialization has competitive performance as that trained from scratch. However, the training process is greatly boosted with 21K pre-trained initialization.
